# Pedicle screw system with telescoping function in surgical treatment for osteoporotic spine

**DOI:** 10.1186/1748-7161-10-S1-P27

**Published:** 2015-01-19

**Authors:** Hideo Hosoe, Nobuki Iinuma, Kohei Nishimura, Hiroshi Nakamura, Masaya Sengoku, Hiroyuki Tanahashi, Hidehiko Nonomura, Tatsuo Yokoi

**Affiliations:** 1 Gifu Prefectural General Medical Center, Japan

## Objective

We will present new modifications for spinal fixation after pedicle subtraction osteotomy (PSO) in aged patients with kyphosis.

## Operative procedure

The pedicle screws in the proximal site of osteotomy are not connected to the rod to permit spinal shortening in the postoperative course. When a connector is used between a pedicle screw and a rod, the connector is not fixed to the rod to allow it to slide on the rod. When a connector is not used, the rod is only placed in the screw head with reinforcement by some sets of cranial sublaminar taping. U-rod is used to not injure the skin when postoperative junctional kyphosis (PJK) occurs.

## Material and method

We investigated 6 cases of posttraumatic kyphosis and a case of degenerative kyphosis. We measured a local kyphotic angle and a distance between the center points of the adjacent endplates at the site of osteotomy.

## Results

Average local kyphotic angle improved from 39 degrees to 6 degrees, and finally increased to 18 degrees. Spinal shortening at the site of osteotomy occurred 8 mm on average (range, three to ten). In two cases without using connectors, kyphosis recurred because sublaminar taping couldn't keep the spine in the correct position.

## Discussion

Patients with osteoporosis have risks of height loss and new vertebral fractures. A good result demands a stable anterior column support. Unexpected excessive spinal shortening by various reasons can lead to instrumentation failures.

Previously we had used rod & wire system as a fixation device after osteotomy for kyphosis with osteoporosis. It has a telescoping function to keep giving the axial load to the anterior column till it attains stability without significant change of sagittal alignment. When we extended the indication of this procedure to the kyphosis or scoliosis with segmental instability, we had to do some re-operations.

We thought up some new modifications of pedicle screw system to compensate the weak points of the rod & wire system. Many cases showed gradual spinal shortening at osteotomy site, keeping a good sagittal alignment. However in some of cases a connector was not used, and the proximal part of instrumented spine gradually separated from the rod. Sublaminar taping seems to be insufficient to keep a good sagittal alignment.

## Conclusion

This new modification of pedicle screw system can be one surgical option for an osteoporotic spine with some morphologically unstable factors.

